# Nonequilibrium sub–10 nm spin-wave soliton formation in FePt nanoparticles

**DOI:** 10.1126/sciadv.abn0523

**Published:** 2022-04-01

**Authors:** Diego Turenne, Alexander Yaroslavtsev, Xiaocui Wang, Vivek Unikandanuni, Igor Vaskivskyi, Michael Schneider, Emmanuelle Jal, Robert Carley, Giuseppe Mercurio, Rafael Gort, Naman Agarwal, Benjamin Van Kuiken, Laurent Mercadier, Justine Schlappa, Loïc Le Guyader, Natalia Gerasimova, Martin Teichmann, David Lomidze, Andrea Castoldi, Dimitri Potorochin, Deepak Mukkattukavil, Jeffrey Brock, Nanna Zhou Hagström, Alexander H. Reid, Xiaozhe Shen, Xijie J. Wang, Pablo Maldonado, Yaroslav Kvashnin, Karel Carva, Jian Wang, Yukiko K. Takahashi, Eric E. Fullerton, Stefan Eisebitt, Peter M. Oppeneer, Serguei Molodtsov, Andreas Scherz, Stefano Bonetti, Ezio Iacocca, Hermann A. Dürr

**Affiliations:** 1 Department of Physics and Astronomy, Uppsala University, 751 20 Uppsala, Sweden.; 2 European XFEL GmbH, Holzkoppel 4, 22869 Schenefeld, Germany.; 3 Department of Physics, Stockholm University, 106 91 Stockholm, Sweden.; 4 Complex Matter Department, Jožef Stefan Institute, Ljubljana, Slovenia.; 5 Max-Born-Institut, Berlin, Germany.; 6 Sorbonne Université, CNRS, Laboratoire de Chimie Physique-Matière et Rayonnement, 75005 Paris, France.; 7 Dipartimento di Elettronica, Informazione e Bioingegneria, Politecnico di Milano, Milano, Italy.; 8 Istituto Nazionale di Fisica Nucleare, Sezione di Milano, Milano, Italy.; 9 Deutsches Elektronen-Synchrotron, 22607 Hamburg, Germany.; 10 Institute of Experimental Physics, Technische Universität Bergakademie Freiberg, 09599 Freiberg, Germany.; 11 Center for Memory and Recording Research, University of California San Diego, 9500 Gilman Drive, La Jolla, CA 92093-0401, USA.; 12 SLAC National Accelerator Laboratory, 2575 Sand Hill Road, Menlo Park, CA 94025, USA.; 13 Faculty of Mathematics and Physics, Department of Condensed Matter Physics, Charles University, Ke Karlovu 5, 121 16 Prague, Czech Republic.; 14 Magnet Materials Unit, National Institute for Materials Science, Tsukuba 305-0047, Japan.; 15 Institut für Optik und Atomare Physik, Technische Universität Berlin, Berlin, Germany.; 16 Department of Molecular Sciences and Nanosystems, Ca’ Foscari University of Venice, 30172 Venice, Italy.; 17 Department of Mathematics, Physics and Electrical Engineering, Northumbria University, Newcastle upon Tyne NE1 8ST, UK.; 18 Center for Magnetism and Magnetic Materials, University of Colorado Colorado Springs, Colorado Springs, CO 80918, USA.

## Abstract

Magnetic nanoparticles such as FePt in the L1_0_ phase are the bedrock of our current data storage technology. As the grains become smaller to keep up with technological demands, the superparamagnetic limit calls for materials with higher magnetocrystalline anisotropy. This, in turn, reduces the magnetic exchange length to just a few nanometers, enabling magnetic structures to be induced within the nanoparticles. Here, we describe the existence of spin-wave solitons, dynamic localized bound states of spin-wave excitations, in FePt nanoparticles. We show with time-resolved x-ray diffraction and micromagnetic modeling that spin-wave solitons of sub–10 nm sizes form out of the demagnetized state following femtosecond laser excitation. The measured soliton spin precession frequency of 0.1 THz positions this system as a platform to develop novel miniature devices.

## Teaser

The smallest magnetic solitons known to date, approaching their fundamental size limit, have been observed in FePt nanoparticles.

## INTRODUCTION

Spin waves are the fundamental excitations in magnetic systems. At low densities, they behave as independent quasiparticles that can mediate solid-state interactions such as superconducting pairing (*1*) or be used to transport information in technology (*2*–*4*). At sufficiently high densities, spin waves can condense into solitons that derive their stability from nonlinear spin precession (*5*–*7*). Generation of spin-wave solitons requires a conservative environment (*8*), where dissipation is matched by excitation, realized within spin-torque nanocontacts (*5*, *6*, *9*). Nonequilibrium conditions via demagnetization with a femtosecond laser pulse provide an alternative generation mechanism (*10*) for topological spin textures, so-called skyrmions (*11*–*13*). So far, the generated spin-wave solitons (*14*–*16*) and skyrmions (*11*–*13*) in materials with perpendicular magnetic anisotropy through femtosecond excitations are too large (several hundreds of nanometers) to be attractive for applications.

**Fig. 1.**
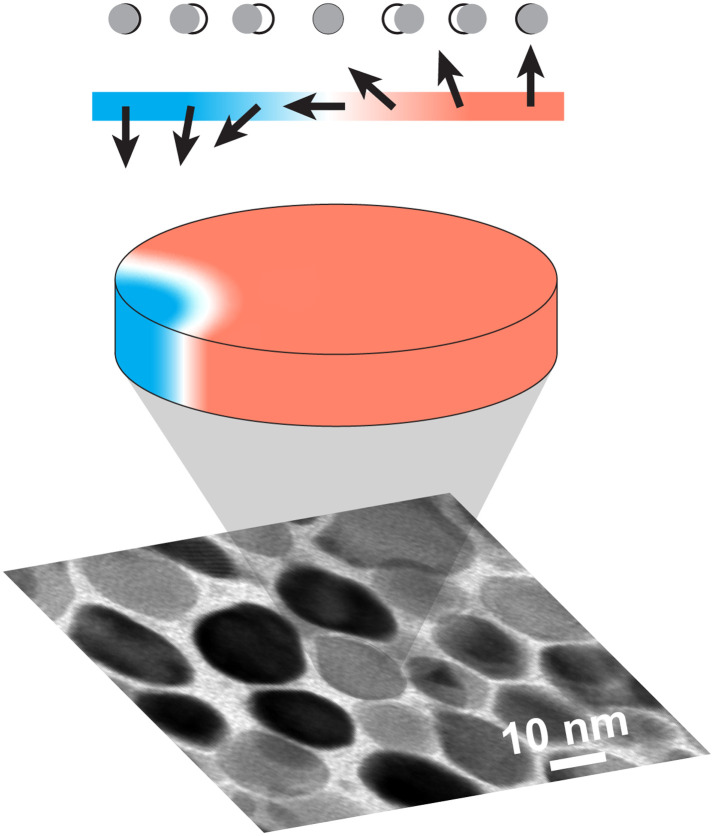
Schematic of FePt sample and magnetoelastic coupling. The bottom panel shows a transmission electron microscopy image of FePt nanoparticles embedded in a C matrix (white). The gray scale represents a spread in crystallographic alignment of the individual nanoparticles. The middle inset shows the average magnetization within one nanoparticle with up (red), down (blue), and in-plane (white) representing the respective magnetization components. The top inset displays the magnetization (colors and arrows) and magnetoelastic atomic displacements (open and gray circles) across a magnetization texture.

Ferromagnetic FePt nanoparticles are natural candidates for supporting spin-wave solitons (*8*) of the ultimate smallest size. The fundamental size limit is given by the so-called exchange length that in FePt is between 1 and 5 nm (*17*) and is thus substantially smaller than typical magnetic nanoparticle sizes (see Fig. 1). The exchange length describes the length scale on which a deviation from a homogeneous magnetic order can occur. It is determined by the competition of the magnetic exchange interaction, which aligns adjacent spins parallel (ferromagnetic) to one another, and the magnetocrystalline anisotropy, which in FePt favors atomic spins oriented along the so-called easy direction of magnetization (for FePt along the cylinder axis in Fig. 1 with red/blue color depicting up/down magnetization components). The magnetocrystalline anisotropy in FePt is extremely large (*17*), leading to small values of the exchange length and, thus, to domain-wall widths on the order of only a few atomic spacings, as schematically shown in the top inset of Fig. 1. This also leads to large magnetoelastic displacements of atoms across the domain wall (see Fig. 1, top inset). In equilibrium, magnetostatics usually favors a single-domain magnetic order in nanoparticles that minimizes both exchange and anisotropy energies (*18*). However, dynamic spin-wave solitons can theoretically exist in nanoparticles, as schematically shown in Fig. 1 for an edge soliton, i.e., one that is pinned to the nanoparticle’s physical boundary.

Here, we show that sub–10 nm spin-wave solitons are self-assembled in FePt nanoparticles following femtosecond laser excitation. Micromagnetic calculations identify the huge FePt magnetocrystalline anisotropy and pinning at the nanoparticle’s boundary as key ingredients for soliton formation. The resulting characteristic soliton dynamics frequencies approach the terahertz regime. They are experimentally verified with time-domain x-ray scattering experiments via the strong FePt magnetoelastic coupling. These results establish a new nanoscale platform for exploring spin-wave solitons with only a few nanometers in size, approaching the theoretical limit of the exchange length that has been elusive to date. This platform also opens the door to markedly miniaturized information processing (*2*–*4*) and possibly bioinspired computing applications (*19*).

## RESULTS

**Fig. 2.**
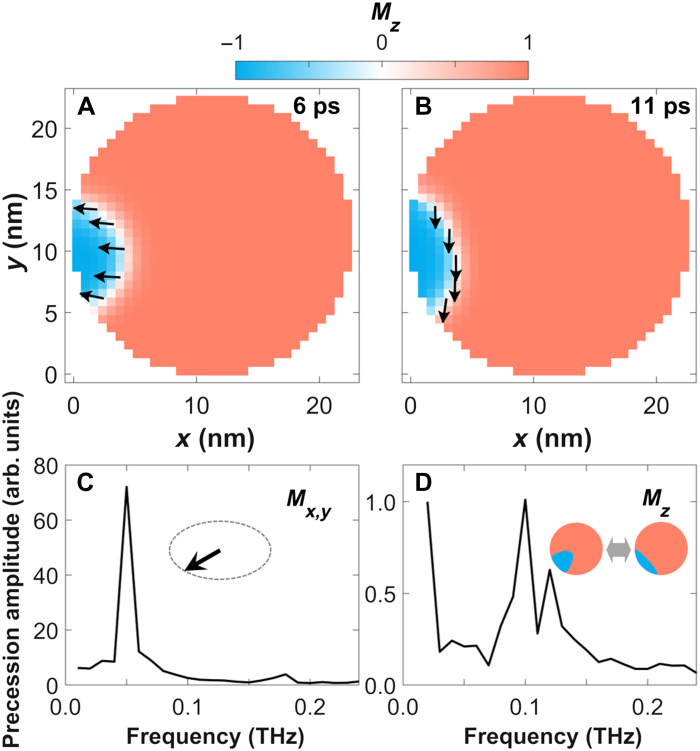
Magnetization dynamics of FePt spin-wave solitons from micromagnetic simulations. (**A** and **B**) Snapshots of the magnetization at 6 and 11 ps. Here, up (red), down (blue), and in-plane (white) represent the respective magnetization components. The in-plane magnetization directions are also indicated by arrows. (**C** and **D**) Frequencies observed for the *M*_*x*,*y*_ and *M_z_* magnetization components obtained via fast Fourier transforms of the full time-dependent simulations.

We generate spin-wave solitons by taking advantage of the approach demonstrated in (*10*) where a randomized spin distribution was induced by an ultrafast quench of the magnetic order after absorption of a femtosecond laser pulse. This nonequilibrium demagnetized state is characterized by large-angle spin fluctuations via excited spin waves. Spin-wave solitons form by localization of long-wavelength spin waves that maintain the total energy of the system at short time scales (*20*). This process is approximately captured by micromagnetic simulations for the small exchange lengths in FePt nanoparticles (see Materials and Methods). We note that micromagnetic simulations are used here to show that steady-state soliton features are observed in the nonequilibrium experimental setting. Figure 2 displays the resulting spin-wave soliton dynamics in a cylindrical FePt nanoparticle with a width of 22.5 nm and a height of 8 nm, which is among the sizes commonly found in our samples (see also movie S1 of the soliton motion). The dynamics is characterized by precession of the in-plane magnetization, depicted in the soliton’s perimeter (white region) in Fig. 2 (A and B). In addition, the spin-wave soliton is quickly attracted to the physical boundary (*21*) and experience both changes in their size (breathing or perimeter modes) (*22*) and translation along the nanoparticle’s edge. These motions are apparent from the two snapshots displayed in Fig. 2 (A and B) (additional snapshots are shown in fig. S4). The characteristic frequencies involved in the in-plane precession and spin-wave soliton motion are shown in Fig. 2 (C and D, respectively). The in-plane precession is characterized by a sharp frequency peak centered around 0.05 THz. The spin-wave soliton motion in Fig. 2D contains two main spectral components: A broad frequency band around 0.10 THz that originates from the spin-wave soliton breathing and a low-frequency contribution <0.02 THz related to coupling of in-plane precession and breathing modes (see the Supplementary Materials).

To date, spin-wave solitons have been detected in extended magnetic thin films by directly imaging the reversed magnetization at the soliton core using x-rays (*14*–*16*). In our case, the much smaller soliton size (see Fig. 2) implies that this is below the resolution limits of typical magnetic x-ray imaging techniques (*23*). We therefore resort to scattering techniques to probe the characteristic magnetization precession and spin-wave soliton breathing frequencies shown in Fig. 2 (C and D). The strong magnetoelastic coupling in FePt (*24*, *25*) provides a convenient means to achieve this goal. Typically, the magnetoelastic force acting on lattice atoms is directly related to the spatial gradient of the magnetoelastic energy change (see Materials and Methods). This implies that variations of the magnetization over very short distances can generate large displacements of lattice atoms as illustrated in the top inset of Fig. 1. The oscillatory nature of the magnetoelastic forces will then drive acoustic lattice waves that propagate throughout the FePt nanoparticles with the speed of sound [4.6 nm/ps for the longitudinal acoustic (LA) mode in FePt]. The localized nature of the spin-wave soliton–induced magnetoelastic forces causes the emitted acoustic waves to be coherent, i.e., the atoms vibrate with a fixed phase relationship. This situation is similar to what has been observed for acoustic strain waves generated at surfaces and interfaces of thin films (*26*, *27*).

**Fig. 3.**
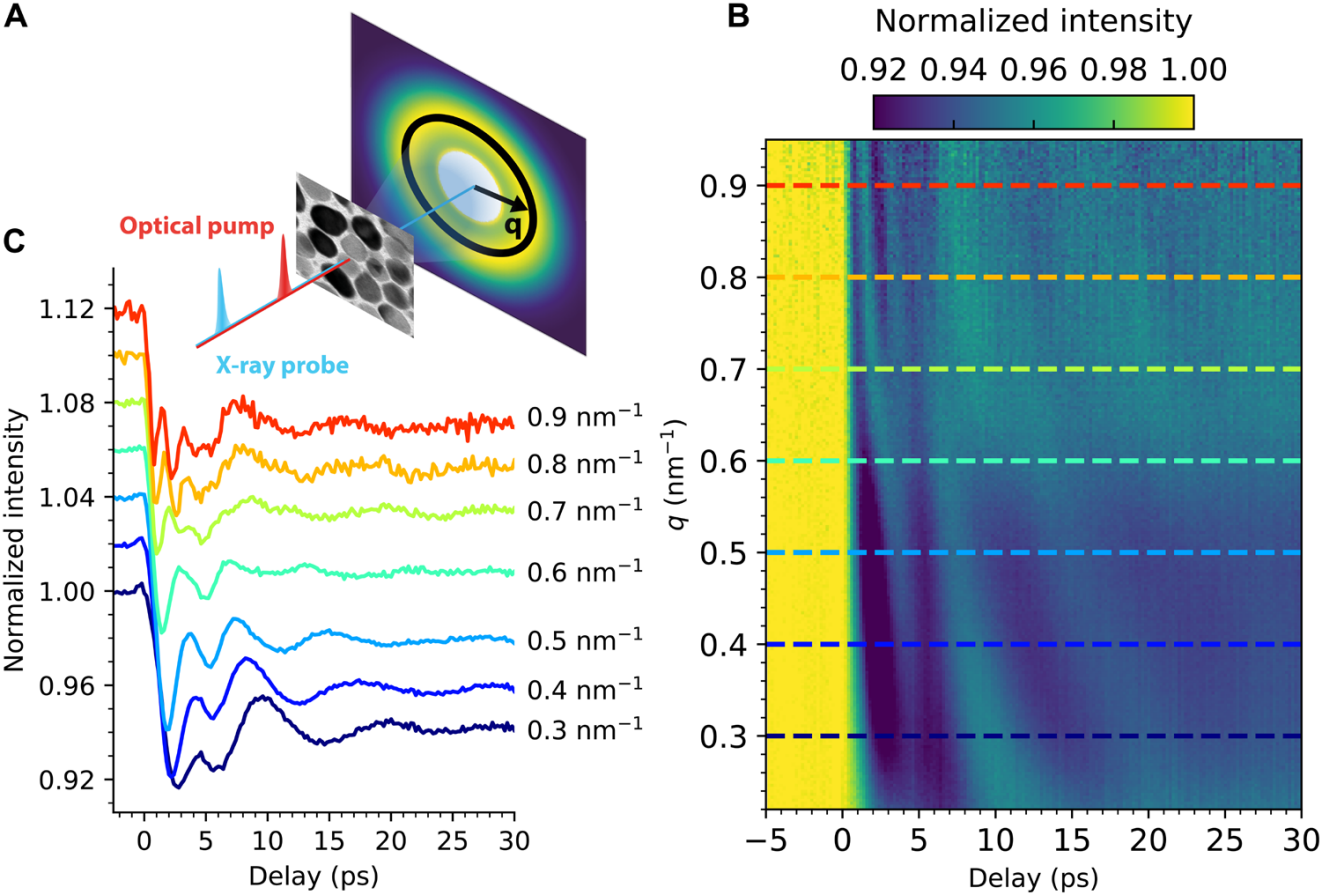
Time-domain measurements of FePt phonons. (**A**) Optical pump x-ray probe experimental geometry with the scattered wave vector, **q**, defined as indicated. (**B**) Time delay map obtained by azimuthally averaging along the black circle in (A) and are normalized to the ground state (negative delay times). (**C**) Linecuts of the time delay map at the indicated values of the wave number, *q*, offset vertically for clarity.

Figure 3 shows the time-domain measurement of the emitted coherent acoustic phonons in FePt nanoparticles. Measurements were performed at the Spectroscopy and Coherent Scattering (SCS) instrument of the European X-ray Free Electron Laser (XFEL) facility (see Materials and Methods). Thirty-femtosecond x-ray pulses of 2500-eV photon energy were scattered as shown schematically in Fig. 3A (marked in blue) with the transferred wave vector, *q*, defined as indicated on the two-dimensional detector. The FePt sample was heated by a 30-fs optical laser pulse (marked in red) intense enough to completely quench the FePt ferromagnetic order. The *q*-dependent scattering signal in Fig. 3 (B and C) is dominated by an initial intensity drop caused by the laser-induced changes of the nanoparticle volume (*24*). The size of this drop is consistent with a 1.4% lattice expansion at our used pump fluence (see Materials and Methods).

**Fig. 4.**
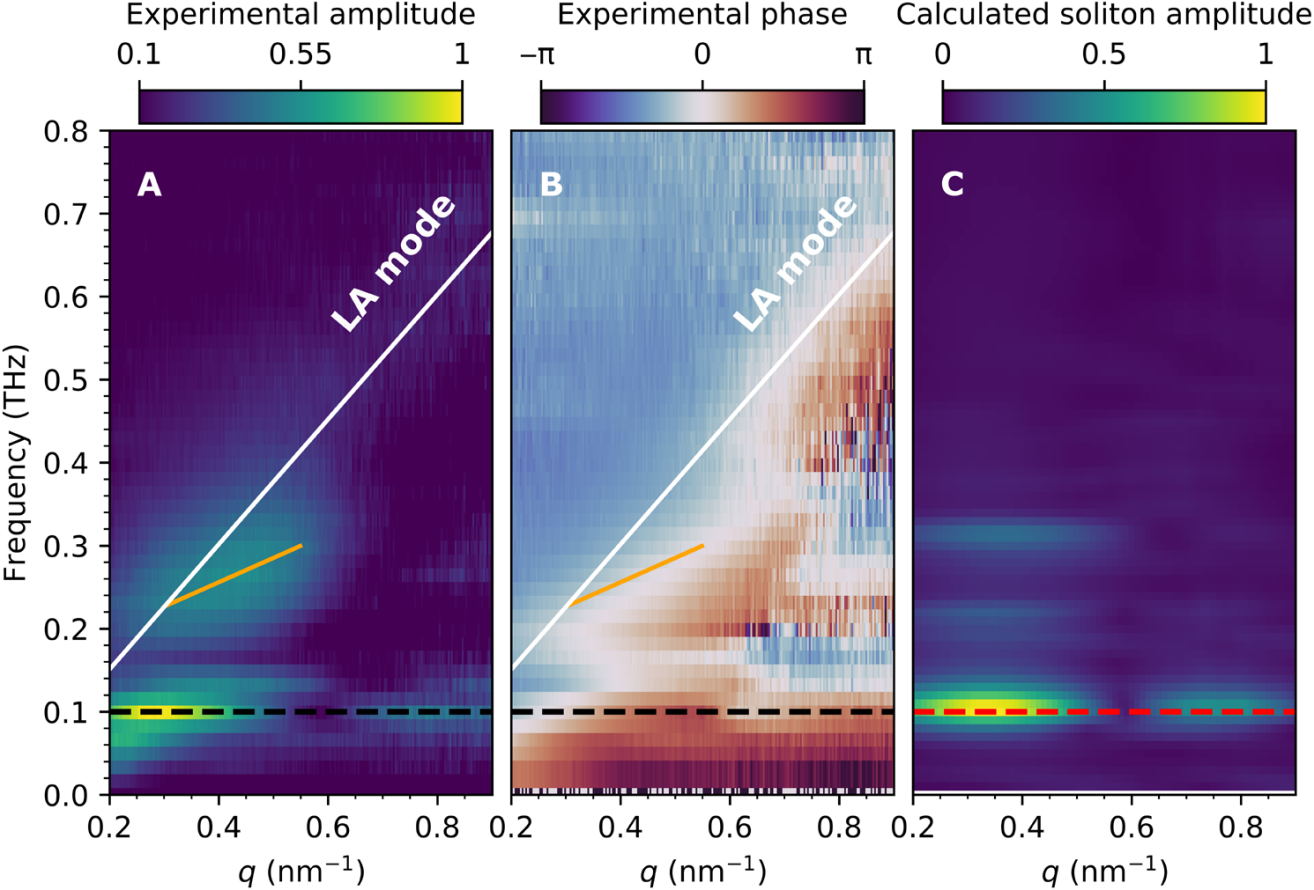
Characteristic frequencies of phonons generated by FePt spin-wave solitons. (**A**) Amplitude and (**B**) phase in the frequency versus wave vector representation of the time-domain data in Fig. 3B. The white lines show the calculated dispersion of the bulk FePt LA phonon mode; the orange and dashed black lines mark the frequencies spin-wave soliton contributions, respectively. (**C**) The amplitude of calculated spin-wave soliton scattering contribution. The dashed red line shows the maximum of calculated spin-wave soliton precession contribution.

Pronounced intensity oscillations are observed at times following the initial intensity drop in Fig. 3 (B and C). These oscillations correspond to coherent phonons composing lattice strain waves as observed previously for thin films (*26*, *27*). The oscillation period displays characteristic variations with *q* that are more clearly visualized in Fourier space. The time-frequency Fourier transform has the functional form *Ae*^*i*φ_0_^, where the determined frequency amplitude, *A*, is shown in Fig. 4A, and the phase, φ_0_, in Fig. 4B.

A feature with a linear dispersion seen in Fig. 4A at high frequencies and large *q* is identified as propagating LA phonons. The white line in Fig. 4A shows the calculated LA mode dispersion (see fig. S7). In analogy to (*26*, *27*), such phonons are excited as strain waves at the nanoparticle boundary and essentially are responsible for expanding the nanoparticle’s volume. However, the most intense mode observed at 0.1 THz has virtually no group velocity.

To clarify the origin of the mode at 0.1 THz, we study the calculated scattering for the spin-wave soliton modes shown in Fig. 2. Details of the calculations are given in Materials and Methods. In brief, we use Eqs. 1 and 2 to obtain the magnetoelastic lattice displacements, **u**, throughout the nanoparticle at each time step of the magnetization dynamics simulation (see movie S1). The displacements, **u**, are the only input for scattering calculations using Eq. 6. We azimuthally average the calculated scattering results to mimic the experimental conditions shown in Fig. 3A. Last, we display in Fig. 4C the frequency amplitudes versus *q* obtained after time-frequency Fourier transform.

**Fig. 5.**
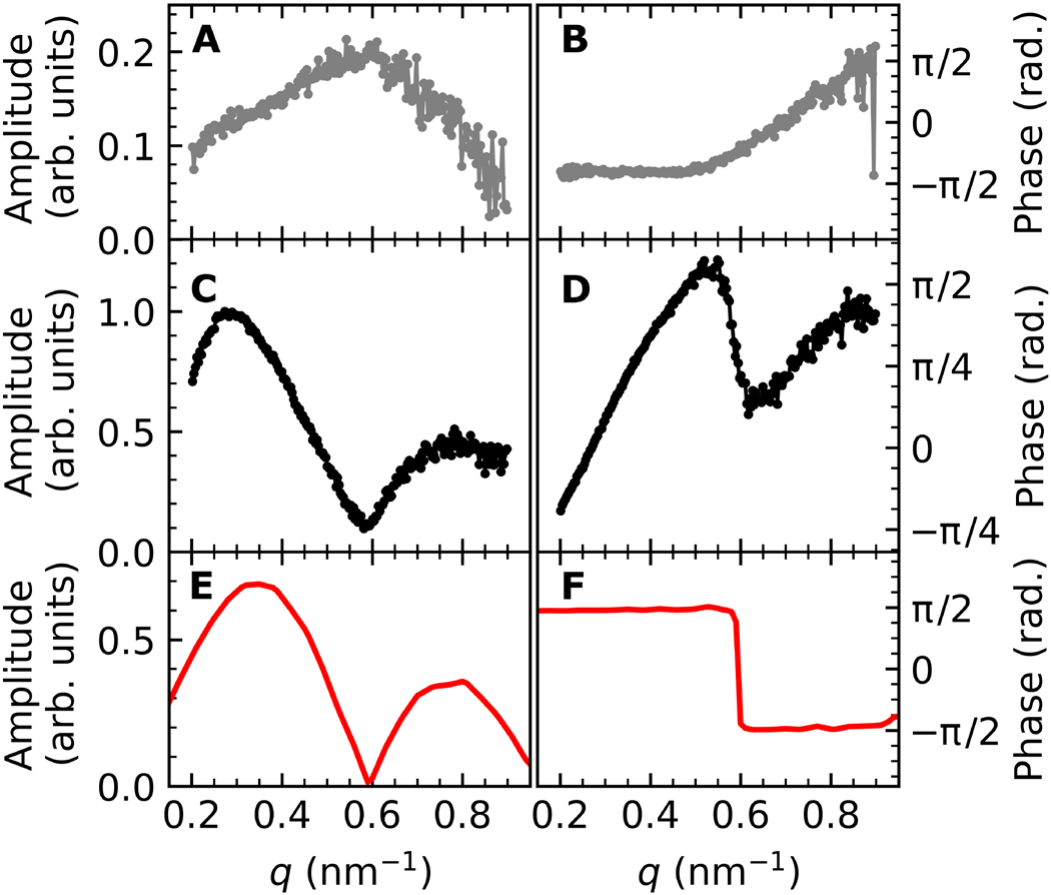
The amplitudes and phases of FePt lattice phonons at selected frequencies. (**A** and **B**) 0.50 THz, dominated by the LA phonon mode; (**C** and **D**) 0.10 THz, where the maximum response of spin-wave soliton precession is observed; and (**E** and **F**) calculated amplitude and phase of the soliton contribution in the scattering at 0.10 THz.

Comparison of Fig. 4 (A and C) allows us to identify the fingerprints of spin-wave solitons in the experimental scattering data. While the high-frequency mode can be assigned to LA strain waves as described above, the frequency bands between 0.2 and 0.3 THz and especially the dominant mode at 0.1 THz are well reproduced by scattering from spin-wave solitons. The *q* dependence of scattering amplitudes and phases for LA and spin-wave soliton modes are compared in Fig. 5 and will be discussed in the following section.

We would lastly like to point out that the observation of coherent scattering fingerprints from spin-wave solitons is noteworthy in itself, since our sample consists of many nanoparticles that would contain solitons. If spin-wave solitons nucleate at different spatial positions within the nanoparticles for each pump-probe cycle or for each nanoparticle, then the net macroscopic coherent scattering would be negligible. We can therefore conclude that the observed coherent scattering signal from a granular alloy implies a nearly deterministic spin-wave soliton nucleation at the perimeters of the nanoparticles.

## DISCUSSION

The modes observed in Fig. 4 with frequencies of 0.2 to 0.3 THz and especially the even more intense feature at 0.10 THz do not agree with the expected LA mode dispersion relation (white line). We can rule out that these modes are associated with transverse acoustic (TA) modes (shown in fig. S7) as the transverse polarization cannot be detected in our experimental geometry (*26*, *27*). In addition, other optical lattice modes have very different frequencies in FePt (*24*) outside the range shown in Fig. 4. The coupling of spin waves to phonons has been observed in the frequency (*28*) and time domain (*29*). However, in FePt, the lowest-energy spin-wave mode is energetically located above 0.69 THz (see fig. S7), and its possible coupling to phonons would result in a very different frequency fingerprint than that observed here. We note that ferromagnetic resonance (FMR) modes observed experimentally between 0.24 and 0.28 THz (*30*) cannot magnetoelastically couple to phonons, as the oscillation amplitude of these FMR modes is nearly homogeneous across the nanoparticle.

Figure 5 (A to D) displays slices along the *q* axis through the scattering amplitudes and phases of Fig. 4 (A and B) at the selected frequencies 0.50 and 0.10 THz. At 0.50 THz (Fig. 5, A and B), the scattering amplitude in Fig. 5A is characterized by a single peak at *q* = 0.57 nm^−1^, which agrees well with that expected from the theoretical LA mode dispersion (Fig. 4A). The observed full width at half maximum, Δ*q* = 0.3 nm^−1^, corresponds to a frequency broadening of Δν = 0.2 THz, which implies that the LA strain waves are heavily damped, as observed in the time-domain measurements of Fig. 3 (B and C). In the simple picture of a driven harmonic oscillator, the LA mode phase should vary from 0 to π when the driving frequency is swept across the LA resonance frequency. A substantial part of this phase characteristics is observed in Fig. 5B. At resonance, the phase is close to zero, while at low *q* values, which corresponds to frequencies below resonance, we observe a phase of −π/2. At higher *q* values, the phase starts to approach π/2, although the measured *q* range is insufficient to actually reach this value.

The scattering characteristic from spin-wave solitons is markedly different. Figure 5C shows that the soliton scattering amplitude displays a two-peak structure. This is reproduced by the model shown in Fig. 5E. The model also allows us to assess the origin of these features. In particular, the dip in scattering amplitude observed at *q* = 0.56 nm^−1^ is caused by scattering from the selected nanoparticle size, i.e., smaller (larger) nanoparticles exhibit the dip at larger (smaller) *q* values. However, the relative intensity of the two peaks in the scattering amplitude observed at *q* = 0.29 and 0.79 nm^−1^ is influenced by the soliton size. Solitons of smaller (larger) size will relatively scatter more (less) at larger *q* values. The good agreement between measured (Fig. 5C) and calculated (Fig. 5E) amplitude *q* dependence of the soliton scattering allows us to conclude that solitons of ~8 nm in size and a 0.05-THz spin precession frequency are formed preferentially in nanoparticles with a diameter of 22.5 nm.

Solitons of a size slightly different to the ~8 nm shown in Fig. 5E should have frequencies that differ from the 0.1-THz magnetoelastic lattice motion driven by the 0.05-THz soliton spin precession. This may explain the broadening along the frequency axis observed around the 0.1-THz amplitude maximum in Fig. 4A. A closer inspection of linecuts along the *q* axis for different frequencies displays different *q* dependencies. This is especially apparent at 0.13 THz, where both the low *q* amplitude maximum and the amplitude dip occur at larger *q* values compared to 0.10 THz. We modeled this behavior in micromagnetic simulations for different particle sizes and obtained good agreement with the experiment for ~7-nm solitons (and a 0.065-THz spin precession frequency) in 19-nm nanoparticles. The amplitude scattering observed for frequencies below 0.1 THz indicates the existence of larger solitons in larger nanoparticles. We did not attempt to model this behavior in more detail because of the limited *q* range of the data. However, the 0.1-THz amplitude maximum clearly indicates that the ~8-nm soliton size residing in 22.5-nm particles is the most abundant one in our samples.

The driving forces of the coherent lattice modes observed in Fig. 4 can be assessed by considering the phase relationship between these modes and the LA phonons. The phase, φ_0_, of coherent oscillations in the time domain (Fig. 3, B and C) describes the temporal offset with which the individual modes oscillate (see Materials and Methods). Figure 4B shows the phase versus *q* plot of the Fourier-transformed data from Fig. 3B. The peak positions visible in the amplitude plot of Fig. 4A are marked by the same lines also shown in Fig. 4B. The phase of the LA mode (for *q* > 0.6 nm^−1^, i.e., where only the LA mode is clearly visible) is identical to that of the frequency band between 0.2 and 0.3 THz within the experimental error of ±0.3 radians.

However, the relative phase of the 0.10-THz mode is significantly different throughout the *q* range (0.2 to 0.5 nm^−1^), where it is visible. The dominant feature in the calculated soliton scattering phase is a shift in Fig. 5F from +π/2 to −π/2 at a wave vector, *q* = 0.6 nm^−1^, corresponding to the dip in scattering amplitude in Fig. 5E. It thus implies a zero crossing of the calculated scattering amplitude. This feature is also clearly visible in the experimental phase values of Fig. 5D. However, experimentally, the zero crossing does occur with a phase offset that is close to π/3. The measured phase (Fig. 5D) can be described by the calculated phase (Fig. 5F) sitting on a monotonously rising offset with increasing *q*. We surmise that this offset is due to the superposition of LA and soliton resonances in the displayed *q* range. Note that such a superposition will affect amplitude and phase differently. This makes a detailed analysis of the 0.10-THz spin-wave soliton phase more difficult than that of its scattering amplitude. This is largely due to the fact that phase changes observed along the frequency axis will extend far beyond the soliton resonance frequency in stark contrast to the phase originating from a relatively narrow amplitude peak. Consequently, a detailed modeling requires knowledge of the soliton size distribution in the sample. Rather than introducing additional fit parameters to account for the soliton size distribution, we limit the discussion here to the features mentioned above.

Our data also allow to estimate the nucleation time of spin-wave solitons. The LA phonons are generated by the laser-induced lattice expansion that starts at the nanoparticle boundary. Their oscillatory lattice displacements composing the propagating strain wave essentially commence with the arrival of the pump laser pulse (*25*, *26*). Also, the 0.2- to 0.3-THz modes start oscillating with the same phase, i.e., at the arrival time of the pump laser pulse. However, the 0.10-THz mode displays a phase lag (up to π/3) relative to the LA mode. If we assume that LA and 0.10-THz modes originate in similar regions of the nanoparticles, i.e., close to the nanoparticle boundaries, then we can express the phase difference as a time delay, which is given by the phase difference divided by the mode frequency as $\frac{{\Delta \varphi }_{0}}{2\pi \nu }\sim 1.7\;\text{ps}$. Note that the sign between LA and 0.10-THz modes demonstrates that the latter starts oscillating ~1.7 ps later. We can, therefore, assign this value to the time it takes a spin-wave soliton to form out of the laser-demagnetized state.

The calculations in Fig. 4C also show soliton-related frequency features between 0.2 and 0.3 THz. These are due to magnetoelastic frequency mixing (for details, see the Supplementary Materials) between the frequency-doubled in-plane magnetization precession (Fig. 2C) and the spin-wave soliton breathing (Fig. 2D). It is tempting to assign them to the observed frequency band that is slightly blue shifted with increasing wave vector (marked with orange lines in Fig. 4). However, the observed zero phase difference relative to the LA modes in Fig. 4B argues for a strain wave–related origin of this frequency band. It is conceivable that strain waves are actually driving part of the soliton motion through magnetoelastically coupling back to the soliton magnetic dynamics. It has been shown that coherent elastic waves can drive spin precession modes in microstructures (*28*). The microscopic origin is the effective magnetic field generated by magnetoelastic coupling (*28*). In our case, the significantly larger strain wave amplitudes would lead to effective magnetic fields of several tesla that could especially influence the *M_z_* magnetization dynamics responsible for the soliton breathing mode (see Fig. 2D). Such a mechanism could also explain the dispersion seen in the 0.2- to 0.3-THz mode (orange lines in Fig. 4), since the strain wave propagation will naturally depend on the size of the nanoparticle that is selected by the transferred wave vector, *q*. However, the detailed modeling of this behavior is beyond the scope of the present paper.

Our results show conclusively that spin wave solitons form in FePt nanoparticles of the demagnetized nonequilibrium state following heating with a femtosecond optical laser pulse. We identify the coherent phonons generated by the spin-wave solitons’ in-plane magnetization precession. The small magnetic exchange length of FePt determines the size of the spin-wave solitons of only several nanometers. This places the observed solitons squarely at the challenging boundary between the atomistic and continuous descriptions of magnetization dynamics (*31*, *32*). Technologically, the writing from up to down in magnetic materials could involve soliton formation before the new equilibrium state possibly with the smallest nanoscale dimensions is stabilized. We anticipate that our work will open up new theoretical and experimental efforts toward the understanding of magnetism at its intrinsic length and time scales, with implications for further scaling strategies in magnetic information storage and processing.

## MATERIALS AND METHODS

### FePt sample growth and characterization

Single-crystalline L1_0_ FePt grains were grown epitaxially onto a single-crystal MgO(001) substrate by cosputtering Fe, Pt, and C (*33*). This resulted in FePt nanoparticles of approximately cylindrical shape with heights of 8 nm and diameters in the range of 5 to 35 nm, with an average of 16 nm (see fig. S1). The FePt nanoparticles form with *a* and *b* crystallographic directions, i.e., the L1_0_ Fe and Pt planes, oriented parallel to the MgO surface. The space in-between the nanoparticles is filled with amorphous carbon. The film was covered by 50 nm of C acting as a heat sink for the pump-probe experiments. Following the sputtering process, the MgO substrate was chemically removed, and the FePt-C films were floated onto copper wire mesh grids with 200-μm-wide openings.

We performed ultrafast electron diffraction from the FePt nanoparticles using the ultrafast electron diffraction (UED) facility at the SLAC National Accelerator Laboratory (*24*). We deduce the FePt lattice expansion along the Fe and Pt atomic planes of the L1_0_ structure (within the sample plane shown in Fig. 3A) as Δ*a*/*a*_0_ = +1.4% ± 0.5% for pump fluences up to 50 mJ/cm^2^ (see fig. S2) in agreement with Fig. 3B.

### Time-resolved x-ray diffraction experiments

The time-resolved tender x-ray diffraction experiments were performed at the SCS instrument of the European XFEL at the photon energy of 2500 eV. The soft x-ray monochromator grating setup in the second diffraction order provides an x-ray bandwidth around 400 meV at 2500 eV and suppresses the higher harmonics. The array of FePt samples were installed on a sample holder that could be moved in all three spatial directions relative to the beam. The x-ray beam was focused on the sample to a spot size of 80 μm using a Kirkpatrick-Baez mirror system. The x-ray fluence on the sample was approximately 0.5 mJ/cm^2^. All measurements were performed at normal x-ray incidence.

The x-ray diffraction patterns were measured using the Deptfet Sensor with Signal Compression (DSSC) detector equipped with miniaturized silicon drift detector pixel arrays (*34*) at a distance of 184 cm from the sample. The DSSC detector has a 1024 × 1024 pixel matrix split into 16 sensors, 128 × 512 pixels each, grouped into four quadrants. The pixels of size 236 × 204 μm^2^ are arranged in the sensors hexagonally. The matrix is covered by a thin Al filter to prevent any optical contamination of the detector image. During the data analysis, the hexagonal pixel array was converted into squares, leading to a negligible error for the count conserving transformation. A mask was applied to the measured patterns to exclude signals from “bad” pixels and residual stray light from upstream beamline elements. The incoming x-ray pulse energy was measured with the x-ray gas monitor (XGM) detector. This value was used for the normalization of diffraction patterns obtained for each x-ray shot.

The pump femtosecond laser used was set to the fundamental wavelength of 800 nm. Laser and x-ray beams are combined in the laser in-coupling chamber, approximately 1 m upstream from the sample. The spatial overlap between the x-ray and laser beams was verified by microscope camera images. The temporal overlap was verified in two stages. Coarse timing was done using the overlap of x-ray and laser signals measured by a photo diode connected to a fast oscilloscope. Fine timing was achieved by measuring the x-ray pump-laser probe reflectivity from a silicon nitride membrane, installed on the sample holder in the same plane as the FePt samples. The laser spot size on the sample was 170 μm, and the pump fluence was 50 mJ/cm^2^. The time resolution of the pump-probe experiment was 60 fs. Experiments were performed at 10-Hz repetition rate using laser-pump x-ray probe pulses and another x-ray pulse arriving approximately 70 μs earlier to probe the initial state of the sample.

The resulting detector images were background substracted, binned according to time delay, and then normalized to the incoming x-ray fluence obtainged from the XGM. To reduce the data size and exploit the symmetries of the system, the scattering patterns were azimuthally integrated, and the intensity as a function of transferred wave vector, *q* (see Fig. 3A), was obtained. The time-dependent scattering pattern was normalized to the ground-state scattering at negative time delays, i.e., the laser-induced differences (with a constant offset of order unity) in the scattering patterns are shown throughout this paper.

We also performed x-ray scattering measurments at SCS with the x-ray energy in resonance with the Fe 2*p*3*d* core-valence resonance at 708 eV. This allowed us to determine the amount of FePt demagnetization in analogy to (*24*, *35*).

### Micromagnetic simulations

The magnetization dynamics of isolated nanoparticles were simulated with the GPU package MuMax 3.9 (*36*). We used a micromagnetic solver to ensure a one-to-one correspondence between the magnetic and magnetoelastic continuum models at a numerical level. We used magnetic parameters for FePt as measured in (*30*): Saturation magnetization *M*_s_ = 950 kA/m, uniaxial anisotropy field μ_0_*H*_k_ = 8.9 T leading to an energy density of *K*_u_ = 4227.5 kJ/m^3^, and Gilbert damping coefficient α = 0.1. The used exchange constant of *A* = 4.1 pJ/m leads to an exchange length *l*_ex_ = 3.1 nm. We used micromagnetic cells with a size of 0.7 nm × 0.7 nm × 0.5 nm, which were found to accurately resolve the dynamics by use of an adaptive Runge-Kutta 45 stepper limited to an upper time step of 1 ps. The simulations presented here pertain to a circular nanoparticle with a diameter of 22.5 nm and a thickness of 8 nm, resulting in a simulation domain of 32 × 32 × 16 = 16,384 cells. This is close to the average nanoparticle size as determined from analysis of a transmission electron microscopy image (see fig. S1). Use of the edge smoothing option in MuMax did not qualitatively affect the results.

Two distinct simulations for a single nanoparticle were performed. First, the remagnetization after ultrafast quenching was modeled as the evolution of the magnetization from a spatially uniform random distribution. This is a crude approximation for the first few to 10 ps, which is better described by atomistic spin dynamics, but yields qualitatively accurate results when the short-wavelength features are relaxed (*10*). The time step in these simulations is typically on the order of tens of attoseconds. We observe the nucleation of solitons akin to magnon localization and coalescence for extended magnetic films with perpendicular magnetic anisotropy (*10*, *13*). A well-defined edge soliton is observed at 80 ps of simulation time. After ~150 ps, the nanoparticle relaxes into a homogeneous magnetization. We note that the nucleation time is not well described by micromagnetic simulations at present. This is because of the artificially high energy of perturbations in a micromagnetic approximation.

To analyze the soliton dynamics, we perform a second set of simulations, where the dissipation is disabled by setting the damping parameter α = 0. We use the soliton relaxed at 80 ps as an initial condition and let the simulation run for 100 ps with a sampling of 50 fs. The goal of this conservative simulation is to numerically extract the soliton modes (breathing, motion, and perimeter in-plane magnetization precession) by Fourier analysis. With the used sampling and simulation time, we obtain a spectral resolution of 10 GHz and an upper frequency of 10 THz. From our simulations, we have estimated that the soliton lifetime in a single particle is on the order of 100 ns. For an array of nanoparticles based on a 1000-nm × 1000-nm experimental image of the sample, stray fields stabilize antiparallel states, and solitons were observed up to 100 ns without a clear decay.

### Magnetoelastic coupling and lattice dynamics calculations

To evaluate the response of the FePt atomic structure to the presence of spin-wave soliton, we performed magnetoelastic calculations using the results of micromagnetic simulations as an input. The spatially localized spin-wave soliton magnetization dynamics causes a strong magnetoelastic force, **f**_mel_, acting on the lattice atom displacements, **u**, via (*37*)

$$\rho \frac{\partial^{2}u}{{\partial t}^{2}}+\frac{2\rho }{\tau }\frac{\partial u}{\partial t}=\nabla \sigma +f_{\text{mel}}$$ (1)

Here, ρ is the mass density, τ is a damping time constant, **σ** is the stress tensor, and ∇**σ** is the elastic force per unit volume, which defines the elastic properties of material. It is determined by the elastic stiffness constants given in Table 1 and by the elastic strains in the lattice (*37*). We have derived the expression of the magnetoelastic force for the tetragonal lattice as (*38*, *39*)

$$\begin{array}{c} f_{\text{mel}}=\frac{1}{M_{0}^{2}}[\begin{array}{c} b_{3}\frac{\partial M_{x}^{2}}{\partial x}\\ b_{3}\frac{\partial M_{y}^{2}}{\partial y}\\ b_{22}\frac{\partial M_{z}^{2}}{\partial z} \end{array}]+\frac{1}{2M_{0}^{2}}[\begin{array}{c} (b_{3}+2b_{21})\frac{\partial M_{z}^{2}}{\partial x}\\ (b_{3}+2b_{21})\frac{\partial M_{z}^{2}}{\partial y}\\ 0 \end{array}]+\\ \frac{1}{M_{0}^{2}}[\begin{array}{c} b_{3}^{\prime }\frac{\partial }{\partial y}(M_{x}M_{y})+b_{4}\frac{\partial }{\partial z}(M_{x}M_{z})\\ b_{3}^{\prime }\frac{\partial }{\partial x}(M_{y}M_{x})+b_{4}\frac{\partial }{\partial z}(M_{y}M_{z})\\ b_{4}\frac{\partial }{\partial x}(M_{z}M_{x})+b_{4}\frac{\partial }{\partial y}(M_{z}M_{y}) \end{array}] \end{array}$$ (2)

where *M*_*x*, *y*, *z*_ are the components of the magnetization vector **M**, *M*_0_ is its size, and $b_{21},b_{22},b_{3},b_{3}^{\prime },\;\text{and}\;b_{4}$ are tetragonal magnetoelastic coupling parameters. Density functional (DFT)–based high-throughput magnetoelastic properties calculations (*38*) were used to compute the magnetoelastic parameters given in Table 1.

**Table 1.** Elastic and magnetoelastic coupling parameters for the tetragonal L1_0_ FePt phase.

**The elastic stiffness tensor** **constants (GPa)**		**The magnetoelastic coupling** **constants (GPa)**	
*C*_1111_	254.8	*b*_21_	0.22
*C*_1212_	105.8	*b*_22_	0.08
*C*_1313_	117.7	*b*_3_	0.10
*C*_1122_	142.8	*b*′_3_	0.43
*C*_1133_	151.2	*b*_4_	−0.05
*C*_3333_	318.8		

The equation of motion, Eq. 1, was solved numerically in the three-dimensional Cartesian grid (similar as in the micromagnetic simulation) using the standard second order “leapfrog” algorithm from the central differences. The dissipation term with τ = 5 ps was included in Eq. 1 to address, in the generalized form, the damping of magnetoelastically induced lattice vibrations via transmission through the nanoparticle boundary into the carbon matrix and other possible mechanisms. From the Fourier analysis of atomic displacements, the characteristic frequencies of lattice vibrations were obtained and are compared to the experimental results in Fig. 4. In general, the magnetoelastic coupling results in a doubling of the soliton large-angle precession mode frequencies, since the force Eq. 2 contains the products of various magnetization components. In turn, the smaller soliton breathing mode amplitudes can be treated in a linearized way. More details are given in the Supplementary Materials.

### X-ray diffraction from coherent phonons in FePt nanoparticles

The scattering intensity at a transferred wave vector **q** from a solid can be expressed as (*40*)

$$I(q)=I_{e}{|\sum_{n}f_{n}e^{iq\cdot r_{n}}|}^{2}$$ (3)

where *f_n_* are the atomic scattering factors for atom *n*, and **r***_n_* are the atomic position vectors. Typically *I*_e_ describes scattering from an individual electron (*40*). However, in our experimental geometry, *I*_e_ also describes the x-ray transmission through the sample (*41*).

Equation 3 can be used to estimate the change in scattering intensity upon lattice expansion following laser heating (see Fig. 3). The atomic scattering factors, *f_n_*, are given by the tabulated optical constants (*42*) that scale inversely proprtional to the lattice unit cell volume, i.e., the atomic density. When the unit cell volume increases because of laser heating, the scattering intensity changes inversely proprtional to it. We note that in our experimental geometry, the incoming x-ray beam averages over the spatial coordinate perpendicular to the sample plane. As a consequence, we only need to take the unit cell expansion perpendicular to the x-ray incidence direction into account. Using the experimentally determined expansion of 1.4% (see previous methods paragraph), we can explain the observed drop in the scattering intensity by 6.6% ± 2.1% (see Fig. 3C).

Equation 3 is also the starting point to describe diffuse x-ray scattering from thermally and optically excited phonons (*26*, *27*, *40*). Rewriting the absolute square in Eq. 3 as

$$I(q)=I_{e}\sum_{n,n\prime }f_{n}f_{n\prime }\;e^{iq∙(r_{n}^{0}-r_{n\prime }^{0})}\;e^{iq∙(u_{n}-u_{n\prime })}$$ (4)

the atomic displacements, **u***_n_*, around the atomic positions at rest, $r_{n}^{0}$, are then replaced by phonons of wavevector, **k**, and phonon branch, *s*, as (*40*)

$$u_{n}=\text{Re}\;\frac{1}{\sqrt{\mu }}\sum_{k,s}\;a_{k,s}e_{k,s}e^{ik∙r_{n}^{0}-i\omega_{k,s}t+i\varphi_{k,s}}$$ (5)

where μ is the atomic mass, ω_**k**,*s*_ is the phonon frequency, and φ_**k**,*s*_ is a phase factor. *a*_**k**,*s*_ and ***e***_**k**,*s*_ describe phonon amplitude and polarization, respectively. Note that while φ_**k**,*s*_ in thermal equilibrium is random and averages to zero (*40*), in our case, φ_**k**,*s*_ is the same for all phonons as long as they are generated by the same spatially localized force. This can either be the spin-wave solitons described here or coherent lattice strain waves due to lattice expansion starting at the nanoparticle boundary.

To evaluate Eqs. 4 and 5, it is common to expand the term in Eq. 4 containing the atomic displacements, **u**, as $e^{iq∙(u_{n}-u_{n\prime })}$ = 1 + *i***q** ∙ **u***_n_* − *i***q** ∙ **u**_*n*^′^_ + *O*(*u*^2^) . In thermal diffuse scattering, the linear terms average to zero, and therefore, the quadratic terms are used to describe the phonon contributions (*40*). This also applies to time-resolved measurements of incoherently excited phonons (*43*, *44*). Here, we use the linear terms that give rise to scattering from coherent phonon wavepackets as demonstrated for thin films (*26*, *27*). We arrive at the scattering from coherent phonons as

$$\begin{array}{c} I_{1}(q)\propto A(q)\;\text{Im}\sum_{n}\;f_{n}\;e^{iq∙r_{n}^{0}}\;q∙u_{n}=\\ A(q)\;\text{Im}\sum_{n}\;f_{n}\;e^{iq∙r_{n}^{0}}\text{Re}\;\frac{1}{\sqrt{\mu }}\sum_{k,s}a_{k,s}q∙e_{k,s}e^{ik∙r_{n}^{0}-i\omega_{k,s}t+i\varphi_{0}} \end{array}$$ (6)

where $A(q)\propto \sum_{n}f_{n}e^{iq∙r_{n}^{0}}$ is the scattering amplitude from the atoms at rest. *A*(**q**) is a real function for the cylindrical nanoparticles considered here. Equation 6 represents the Fourier transform (*n* summation) over a set of waves propagating in direction, **k**, with constant phase, φ_0_. The term, **q ∙ e**_**k**,s_, implies that phonons with a polarization vector parallel to the scattered wave vector are preferentially detected. It is important to reiterate that the phase term *e*^*i*φ_0_^ in Eq. 6 is characteristic for the force that generates the coherent phonons. This is used in Fig. 4 to differentiate between phonons generated via strain waves at the nanoparticle boundary and phonons generated by spin-wave solitons.

We use Eq. 6 to calculate the scattering pattern from spin-wave solitons contained in cylindrical nanoparticles. The spin-wave soliton magnetization dynamics (see Fig. 2 and fig. S4, A to F) generates a magnetoelastic force that acts on the lattice atoms (see fig. S5A). The corresponding atomic displacements, **u***_n_*, are calculated from Eqs. 1 and 2. In these calculations, we take *A*(**q**) to be constant, i.e., without any **q** dependence. This procedure reflects the normalization of the time-resolved x-ray diffraction measurments by the scattering yield before time zero, i.e., the FePt ground-state configuration. Figure 4C shows the calculated scattering amplitude of the spin-wave soliton mode. In particlular, the *q* characteristics of the spin-wave soliton scattering near 0.10 THz closely resembles the experimental result in Fig. 4A.

### Calculations of FePt phonons and spin waves

The magnon dispersion of bulk FePt was calculated using a DFT-based approach. First, the DFT electronic structure of FePt was computed using the tight-binding linear muffin-tin method within the atomic sphere approximation (*45*). The DFT exchange-correlation potential was described by the local spin density approximation in the parametrization of Vosko *et al.* (*46*). This approach has been used recently to study the atomic magnetic moments on Fe and Pt in FePt (*47*). The magnon spectrum was subsequently computed by mapping the total energy on the Heisenberg model (*48*). The effective pair exchange interactions *J_ij_* of the Heisenberg model that are required were computed using the Liechtenstein formula (*49*).

The computed lowest-energy magnon dispersion of FePt is shown in fig. S6. The magnetocrystalline anisotropy energy (MAE) leads to an upward shift of the spin-wave energy around the Γ point by the MAE (for one FePt unit) by ~0.69 THz (*50*), in good agreement with other calculations (*51*) and inelastic neutron scattering measurements (*52*).

We performed phonon calculations following (*24*). This resulted in values of the speed of sound of 4.6 nm/ps for the LA and 2.6 and 1.7 nm/ps for the TA phonon modes. Frequency dispersions of the phonon modes are shown in fig. S7 together with the lowest-energy magnon mode.
