# Supplementary material for: Nonequilibrium sub–10 nm spin-wave soliton formation in FePt nanoparticles
Source: Sci Adv. 2022 Apr 1;8(13):eabn0523. doi: 10.1126/sciadv.abn0523 (PMC10938569; doi:10.1126/sciadv.abn0523)
Supplement: Supplementary file 1 — Supplementary Text Figs. S1 to S7 Table S1 [file sciadv.abn0523_sm.pdf]

Science Advances
AAAS

Supplementary Materials for

Nonequilibrium sub–10 nm spin-wave soliton formation in FePt nanoparticles

Diego Turenne, Alexander Yaroslavtsev, Xiaocui Wang, Vivek Unikandanuni, Igor Vaskivskyi, Michael Schneider, Emmanuelle Jal, Robert Carley, Giuseppe Mercurio, Rafael Gort, Naman Agarwal, Benjamin Van Kuiken, Laurent Mercadier, Justine Schlappa, Loïc Le Guyader, Natalia Gerasimova, Martin Teichmann, David Lomidze, Andrea Castoldi, Dimitri Potorochin, Deepak Mukkattukavil, Jeffrey Brock, Nanna Zhou Hagström, Alexander H. Reid, Xiaozhe Shen, Xijie J. Wang, Pablo Maldonado, Yaroslav Kvashnin, Karel Carva, Jian Wang, Yukiko K. Takahashi, Eric E. Fullerton, Stefan Eisebitt, Peter M. Oppeneer, Serguei Molodtsov, Andreas Scherz, Stefano Bonetti, Ezio Iacocca, Hermann A. Dürr*

*Corresponding author. Email: hermann.durr@physics.uu.se

Published 1 April 2022, *Sci. Adv.* **8**, eabn0523 (2022)
DOI: 10.1126/sciadv.abn0523

The PDF file includes:

Supplementary Text
Figs. S1 to S7
Table S1
Legend for movie S1

Other Supplementary Material for this manuscript includes the following:

Movie S1

A model for FePt magneto-elastic coupling

This section describes in more detail the magneto-elastic coupling outlined in the methods section. The response of the FePt lattice structure to the presence of a spin-wave soliton in the nanoparticle was investigated using the magneto-elastic coupling formalism *(36)*, that describes the influence of magnetization dynamics on lattice atom displacements, **u**, in the material. In Cartesian coordinates, **x**, the magneto-elastic energy density for the tetragonal FePt lattice is given by the eq. (1) *(37,38)*:

$$\varepsilon_{mel}^{tet} = b_{11}\left(\varepsilon_{xx} + \varepsilon_{yy}\right) + b_{12}\varepsilon_{zz} + b_{21}\left(\alpha_z^2 - \frac{1}{3}\right)\left(\varepsilon_{xx} + \varepsilon_{yy}\right) + b_{22}\left(\alpha_z^2 - \frac{1}{3}\right)\varepsilon_{zz}$$
$$+\frac{1}{2}b_3\left(\alpha_x^2 - \alpha_y^2\right)\left(\varepsilon_{xx} - \varepsilon_{yy}\right) + 2b_3'\alpha_x\alpha_y\varepsilon_{xy} + 2b_4\left(\alpha_x\alpha_z\varepsilon_{xz} + \alpha_y\alpha_z\varepsilon_{yz}\right), \qquad (1)$$

where $\alpha_{x,y,z}$ are the direction cosines of the magnetization vector, **M**, $\varepsilon_{ij} = \frac{\partial u_i}{\partial x_j}$ are components of the strain tensor and $b_{11}, b_{12}, b_{21}, b_{22}, b_3, b_3', b_4$ are tetragonal magneto-elastic coupling constants. Using the magneto-elastic energy density it is possible to calculate the magneto-elastic force:

$$\mathbf{f}_{mel} = \nabla\left(\frac{\mathrm{d}\varepsilon_{mel}}{\mathrm{d}\varepsilon_{ij}}\right) \qquad (2)$$

For the tetragonal lattice eq. (2) leads to the following expression:

$$\mathbf{f}_{mel}^{tet} = \frac{1}{M_0^2}\begin{bmatrix} b_3\frac{\partial M_x^2}{\partial x} \\ b_3\frac{\partial M_y^2}{\partial y} \\ b_{22}\frac{\partial M_z^2}{\partial z} \end{bmatrix} + \frac{1}{2M_0^2}\begin{bmatrix} (b_3+2b_{21})\frac{\partial M_z^2}{\partial x} \\ (b_3+2b_{21})\frac{\partial M_z^2}{\partial y} \\ 0 \end{bmatrix} + \frac{1}{M_0^2}\begin{bmatrix} b_3'\frac{\partial}{\partial y}(M_xM_y) + b_4\frac{\partial}{\partial z}(M_xM_z) \\ b_3'\frac{\partial}{\partial x}(M_yM_x) + b_4\frac{\partial}{\partial z}(M_yM_z) \\ b_4\frac{\partial}{\partial x}(M_zM_x) + b_4\frac{\partial}{\partial y}(M_zM_y) \end{bmatrix} \qquad (3)$$

In eq. (3) the first and the second terms are isotropic and the third term is the anisotropic component of the magneto-elastic force.

We note that all terms include spatial derivatives of the magnetization. This means that the force is nonzero only if the magnetization is not homogeneous over the nanoparticle volume. This implies that if the length of the magnetization vectors per unit volume does not change, the direction of the magnetization must change across the nanoparticle at any particular moment of time. As a consequence, homogeneous magnetic modes such as ferromagnetic resonance (FMR) cannot produce a considerable magneto-elastic coupling because the magnetic moments of all atoms in the nanoparticle precess as a whole with a similar amplitude and phase. Contrary to FMR, the spin-wave soliton has a well-defined profile that gives rise to strong magneto-elastic forces. These forces are especially large at the spin-wave soliton's perimeter. Here the M_z component reverses sign while $M_{x,y}$ experiences a large-angle in-plane precession.

We calculate the details of the lattice dynamics in FePt nanoparticles by solving the equation of motion for the elastically strained solid with the magneto-elastic force resulting from the soliton magnetization dynamics as a driver for the atomic displacement:

$$\rho\frac{\partial^2\mathbf{u}}{\partial t^2} + \frac{2\rho}{\tau}\frac{\partial\mathbf{u}}{\partial t} = \nabla\boldsymbol{\sigma} + \mathbf{f}_{mel} \qquad (4)$$

where $\mathbf{u}$ is the displacement per unit volume, ρ is the mass density, τ is a damping time constant, $\mathbf{f}_{mel}$ is the magneto-elastic force and $\nabla\boldsymbol{\sigma}$ is the elastic force per unit volume

which defines the elastic properties of material and the phonon spectrum. It is given by the gradient of the three-dimensional stress tensor:

$$\sigma_{ij} = \sum_{k=1}^{3} \sum_{l=1}^{3} C_{ijkl} \varepsilon_{kl}, \qquad (5)$$

where C_{ijkl} are the components of the fourth-rank tensor of elastic stiffness parameters.

We calculate the magneto-elastic force $\mathbf{f}_{mel}$ using the expression eq. (3) with the results of micromagnetic simulation for an 8 nm high and 22.5 nm wide cylindrical nanoparticle as input. This is among the nanoparticle sizes commonly found in the sample. The equation of motion (4) was solved numerically in the 3-dimensional Cartesian grid using the standard second order “leapfrog” algorithm from the central differences. We used the same grid spacing as in the micromagnetic simulation: 0.7 nm in x, y and 0.5 nm in z directions. The time interval of simulation was 100 ps with the time step 50 fs. The initial lattice displacements were assumed to be zero, $\mathbf{u}_0 = 0$. For simplicity we have used the Dirichlet boundary condition $\mathbf{u}_{boundary} = 0$. The dissipation term with $\tau = 5$ ps was included in the eq. (4) to address, in the generalized form, the damping of magneto-elastically induced lattice vibrations via transmission through the nanoparticle boundary into the carbon matrix and other possible mechanisms.

Figure S4 illustrates the spin-wave soliton magnetization dynamics obtained from micromagnetic simulations described in the main paper (see methods section). The 22.5 nm nanoparticle displays a specific soliton mode with a frequency of 0.05 THz (Fig. S4F) for the in-plane magnetization components M_x and M_y. This mode is best observed as a precession of the magnetization along the soliton’s perimeter (marked in white in Figs. S4A-E). At the same time the dynamics of the out-of-plane M_z component shows a lower-frequency peak near 0.10 THz that is related to the soliton breathing, i.e. periodic changes of its size and shape. In Figs. S4A-E, snapshots of the magnetization state demonstrate the spin-wave soliton precession and breathing dynamics. While the magnetization at the perimeter makes one full 360° rotation, the soliton shape changes about twice as fast. The soliton breathing can be clearly seen as the squeezed and elongated (blue) shapes in Figs. S4A-E (see also Movie S1).

Figure S5A shows the spectra of the magneto-elastic force calculated as the time-frequency Fourier transform of the x, y, z force components in eq. (3) averaged over the particle’s volume. The used FePt magneto-elastic coupling parameters are shown in Table 1 in the methods and materials section of the main paper. We see a strong peak at 0.10 THz and a weaker broader band between 0.2-0.3 THz. The former is caused mainly by the precession of the magnetization along the soliton’s perimeter, while the latter corresponds to the magneto-elastic frequency mixing between the frequency-doubled in-plane magnetization precession and the spin-wave soliton breathing.

This assignment can be explained by considering a simplified model of the soliton magnetization precession and breathing modes. The x, y, and z components of magnetization near the soliton perimeter have the following form:

$$M_x(x,y) = M_0 \sin\theta_0\big(r(x,y)\big)\cos(\omega t)$$
$$M_y(x,y) = M_0 \sin\theta_0\big(r(x,y)\big)\sin(\omega t)$$
$$M_z(x,y) = M_0 \cos\theta_0\big(r(x,y)\big), \qquad (6)$$

where ω is the frequency of the soliton mode observed in M_x and M_y (Fig. S4F), that corresponds to the magnetization precession; while $\theta_0\big(r(x,y)\big)$ is the spatial dependence of the polar magnetization angle for a spherical spin-wave soliton described in ref. *(7)*. θ_0 is zero at the soliton perimeter but its detailed shape is only accessible to numerical calculations *(7)*. We will use θ_0 here to parametrize the soliton shape. To address the spin-wave soliton breathing and it's coupling to phonons, the substitution can be made:

$$x \rightarrow x + \Delta x \cos\omega' t, \qquad (7)$$

where ω' is the soliton breathing frequency observed mainly in the M_z component (Fig. S4F) and Δx describes the breathing motion of the soliton perimeter which is "small" compared to the soliton size. Substituting the magnetization components calculated using eqs. (6) and (7) into eq. (3) the following approximate terms of the magneto-elastic force x component can be derived:

$$f_{x1.1} = \frac{b_3}{2}\sin 2\theta_0 \frac{\partial\theta_0}{\partial r}\,\frac{(1+\cos 2\omega t)(x+\Delta x\cos\omega' t)}{r}$$
$$f_{x1.2} = -\frac{(b_3+2b_{21})}{2}\sin 2\theta_0 \frac{\partial\theta_0}{\partial r}\,\frac{(x+\Delta x\cos\omega' t)}{r}$$
$$f_{x2} = \frac{b_3'}{2}\sin 2\theta_0 \frac{\partial\theta_0}{\partial r}\,\frac{\sin 2\omega t\,(y-\Delta x\cos\omega' t)}{r}$$
$$f_{x3} = b_4\cos 2\theta_0 \frac{\partial\theta_0}{\partial z}\cos\omega t \qquad (8)$$

In eqs. (8) we can notice the terms which contain the phases $2\omega t$ and $\omega' t$. Since the micromagnetic simulations of a 22.5 nm particle give $\omega \approx 0.05$ THz and $\omega' \approx 0.10$ THz (Fig. S4F), these terms apparently cause the peak in the force frequency spectrum at ~ 0.10 THz (Fig. S5A) as the second harmonic of ω and the fundamental ω' frequency. Eqs. (8) also contain cross-terms with the product of $2\omega t$ and $\omega' t$, that will lead to the addition (and subtraction) of these frequencies together resulting in the frequency band between 0.2-0.3 THz (and very low frequencies contributions that are not resolved experimentally). Thus, two frequency bands in the magneto-elastic force in Fig. S5A can be explained by the terms containing the doubled spin-wave soliton precession and the breathing frequency with $2\omega \approx \omega' \approx 0.10$ THz as well as the cross-terms between them. In other words, the interference between the magneto-elastic interactions caused by the soliton magnetization precession near the soliton perimeter and the soliton breathing produces two bands which differ approximately by a factor of two in frequency.

Furthermore, $f_{x1.1}$ in eq. (8) contain a time-independent term causing a constant force component near the domain wall, which is indeed observed in the full simulation. Finally, the term f_{x3} may produce a linear frequency band near $\omega \approx 0.05$ THz. However, it is not observed in the experimental result because the soliton polar angle θ_0 at the perimeter likely does not change along the z direction, so that the derivative $\frac{\partial \theta_0}{\partial z}$ is close to zero. Also, the magneto-elastic constant b_4 responsible for this coupling is the smallest of all.

Figure S5B shows the calculated frequency spectrum of in-plane atomic displacements averaged over the particle volume. It shows a distinct peak at 0.10 THz and a weaker, broader band between 0.2-0.3 THz. The former corresponds well to the peak of the magneto-elastic force at the same frequency and is caused by the forced harmonic oscillations of the atoms with this frequency. The band at 0.2-0.3 THz overlaps with the band of the magneto-elastic force at the same frequency range and thus it is of induced nature as well. On the whole, the dominating lattice vibration bands at 0.10 and 0.2-0.3 THz, calculated for the 22.5 nm particle, are in good agreement with the soliton scattering contribution in experimental result in Fig. 4A of the main paper.

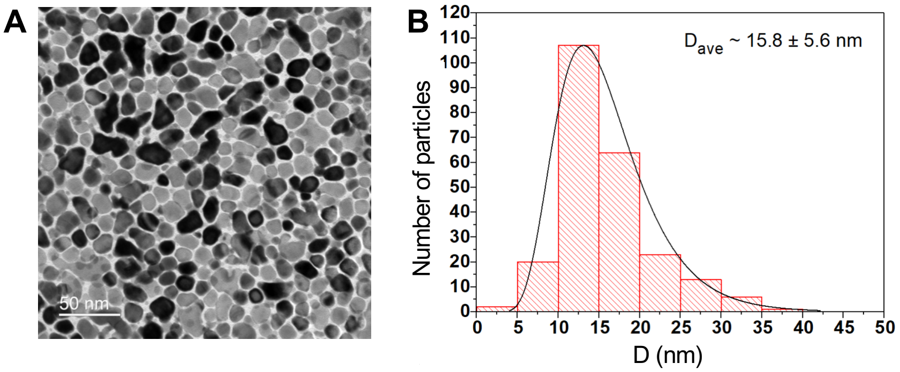

Fig. S1. Nanoparticle size distribution of FePt samples: (**A**), transmission electron microscopy (TEM) image of FePt nanoparticles embedded in a C matrix (white). The different grey-scales visible in the FePt nanoparticles is caused by a spread in crystallographic alignment of the individual nanoparticles. (**B**), distribution of nanoparticle sizes, D. The average particle size evaluated from the TEM image is D_{ave} = 15.8 ± 5.6 nm. The grain size distribution and average grain size were extracted from plane-view bright-field TEM images. More than 1,500 grains were randomly selected for the statistical analysis. The selected grains were covered with masks in the bright-field TEM image and the diameter information was acquired through analysis with the Gatan Microscopy Suite software. The collected data were fitted by a lognormal function to determine the average grain size and its standard deviation (error) to D_{ave} = 15.8 ± 5.6 nm.

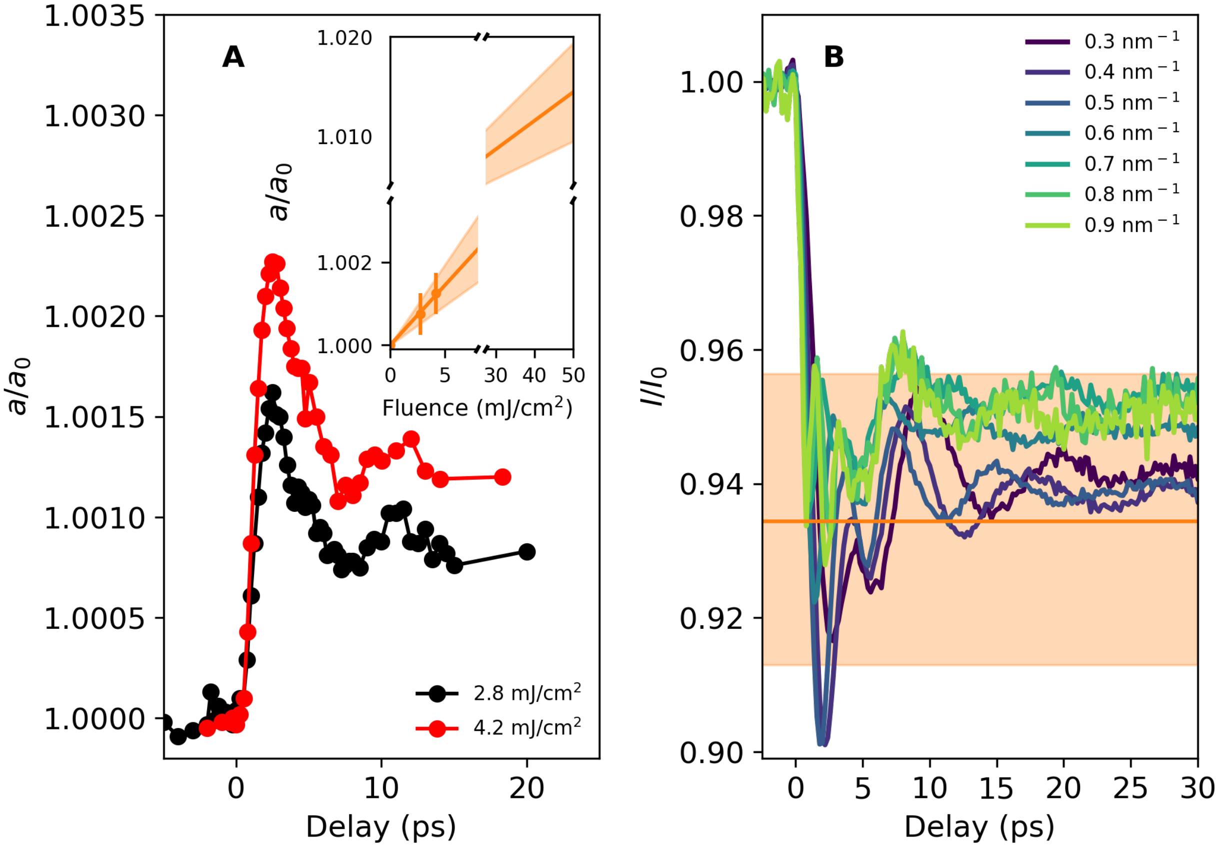

Fig. S2. Effect of the lattice expansion on X-ray scattering contrast: (**A**), Lattice expansion vs. pump-probe time delay for $L1_0$ FePt nanoparticles. Ultrafast electron diffraction (UED) was used to determine the changes of the lattice spacings parallel to the Fe and Pt layers (a-direction) of the $L1_0$ FePt lattice following fs laser excitation with 2.8 mJ/cm^2 (black symbols) and 4.2 mJ/cm^2 (red symbols), the inset shows that linear extrapolation to the fluence used in the main paper results in changes of $\Delta a/a_0 = +\ 1.4\ \% \pm 0.5\ \%$ as obtained for delay times above ~5 ps where the values for a/a_0 are approximately constant. (**B**), scattering-intensity-drop calculated for the 1.4% change in lattice constant (orange line) overlaid on top of the measured X-ray scattering signal. The curves shown are the same as in Fig. 3 but this time they have not been shifted along the y-axis. The experimental results are within the extrapolation error (shaded in orange).

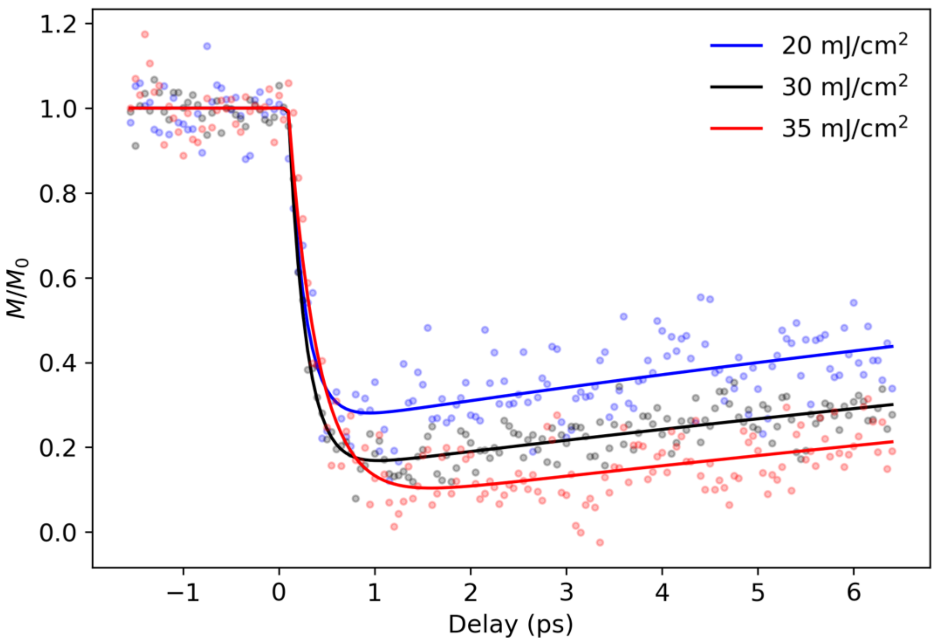

Fig. S3. Ultrafast demagnetization of FePt for the indicated pump fluences. Following refs. *(23,33)* circularly polarized X-rays with energy tuned to the Fe 2*p* – 3*d* core-valence resonance (708 eV) were scattered from the FePt sample in the geometry shown in Fig. 3A. The sample magnetization was oriented (anti)parallel to the X-ray incidence direction by applying a magnetic field of ±350mT. The difference in the scattering yield, integrated over all accessible wavevectors, is a measure of the sample magnetization, M, and is plotted relative to the ground-state magnetization, M_0, measured before arrival of the pump pulse. The measured data (symbols) are fitted with an exponential decay followed by an exponential recovery (lines) of the form, $M/M_0 = 1 + A[exp(-\frac{t-t_0}{\tau_{rm}}) - exp(-\frac{t-t_0}{\tau_{dm}})]$ *(23,33)*. The fit parameters are compiled in Table S1.

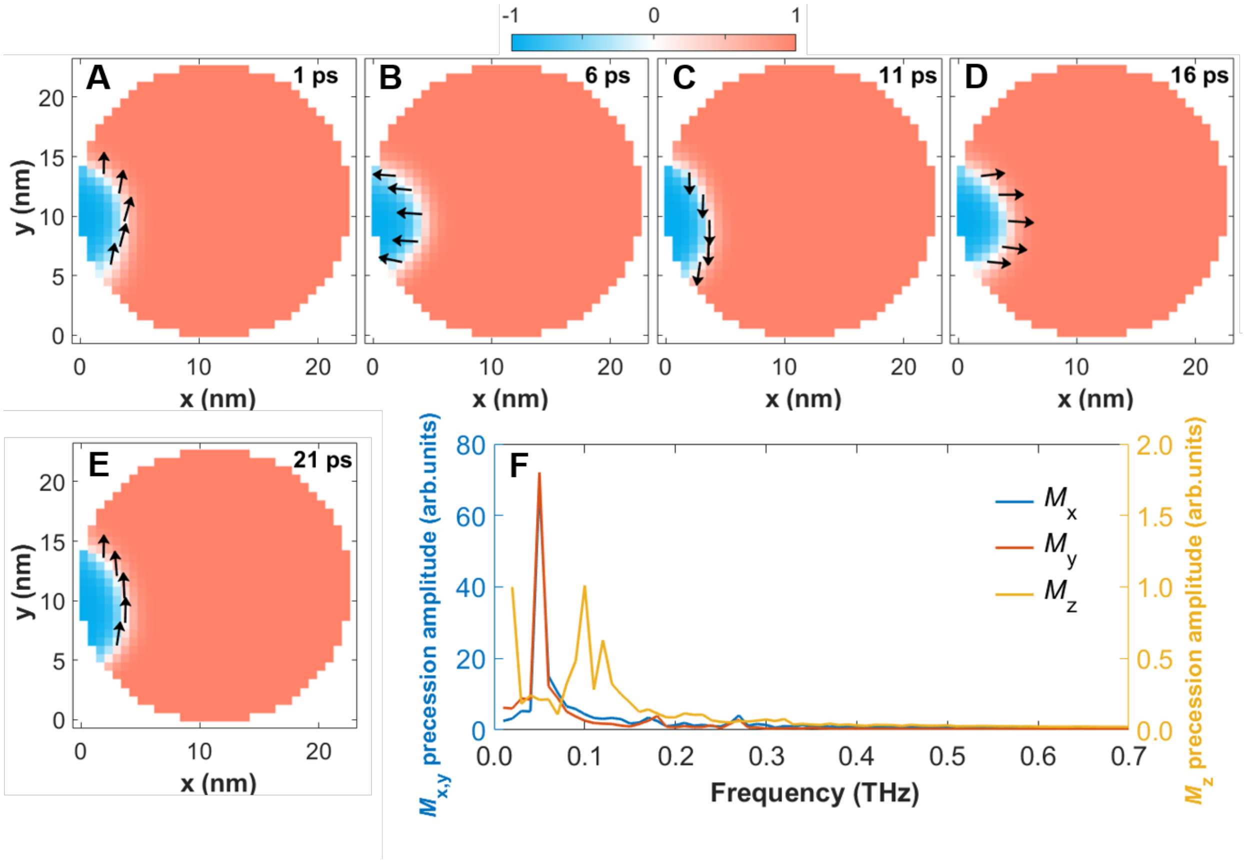

Fig S4. Micromagnetic simulations of spin-wave solitons in nanoparticles. (**A-E**), Snapshots of the micromagnetic simulation showing the correspondence between the two phases of soliton distortions (breathing and motion) and perimeter precession. The colour scale denotes the out-of-plane M_z component. The black arrows show the magnetization in the perimeter that is predominantly oriented in-plane. (**F**), The Fourier transform amplitudes for the M_x, M_y and M_z magnetization components. The frequency of the M_z precession corresponds to the spin-wave soliton breathing and motion.

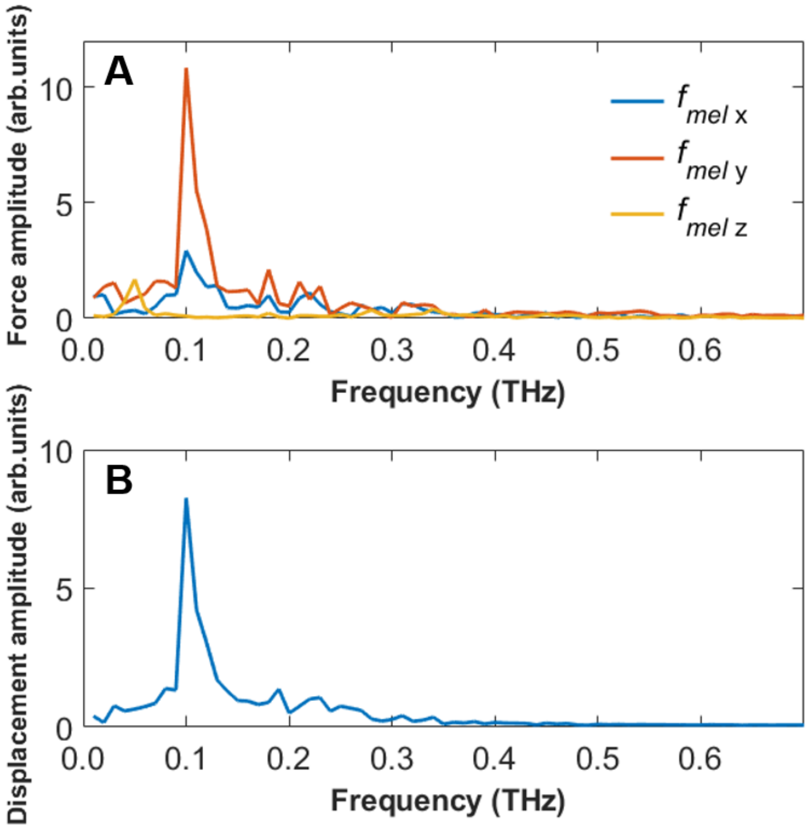

Fig. S5. Results of the numerical magneto-elastic calculations: amplitudes of the Fourier transform of (**A**), magneto-elastic force and (**B**), atomic displacement. Both quantities show two dominating frequency bands at 0.10 and 0.2-0.3 THz.

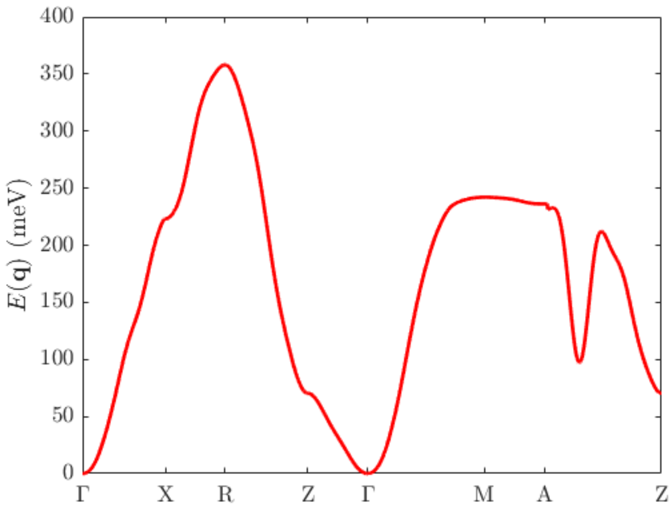

Fig. S6. Calculated FePt spin-waves. *Ab initio* calculated spin-wave dispersion of bulk FePt in the simple tetragonal Brillouin zone with corresponding high-symmetry points as indicated.

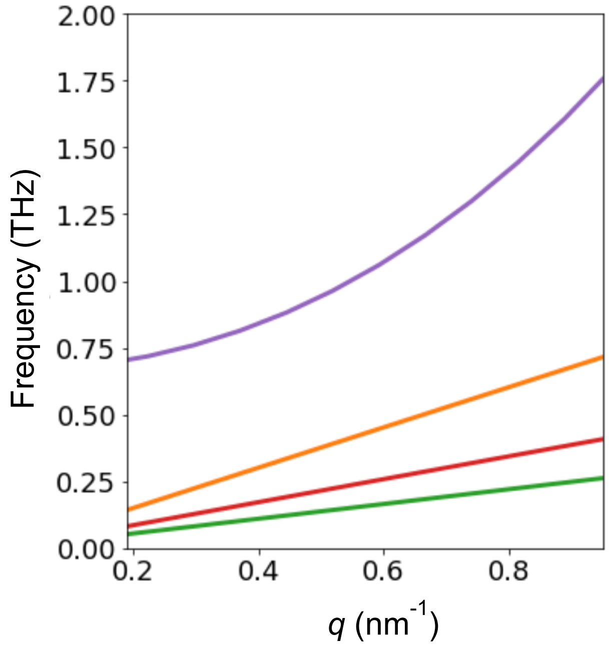

Fig. S7. FePt spin-wave and phonon dispersions. Calculations of magnon (purple line) and phonon (orange: longitudinal acoustic, LA, mode, red, green transverse acoustic, TA, modes) frequencies vs. wavevector, q.

Table S1. The fitted parameters of the ultrafast magnetization dynamics in FePt of the form, $M/M_0 = 1 + A[exp(-\frac{t-t_0}{\tau_{rm}}) - exp(-\frac{t-t_0}{\tau_{dm}})]$ obtained from Fig. S3.

Fluence (mJ/cm^2)	A	τ_{dm} (ps)	τ_{rm} (ps)
20	0.754±0.017	0.176±0.021	21.4±2.9
30	0.863±0.009	0.184±0.010	29.9±2.6
35	0.943±0.018	0.314±0.021	34.5±5.7

Other Supplementary Materials for this manuscript include the following:

Movie S1

Movie S1. Movie of the temporal evolution of the magnetization of the FePt nanoparticle shown in Figs. 2A, B.
